# Assessment and Diagnosis of Down Syndrome Regression Disorder: International Expert Consensus

**DOI:** 10.3389/fneur.2022.940175

**Published:** 2022-07-15

**Authors:** Jonathan D. Santoro, Lina Patel, Ryan Kammeyer, Robyn A. Filipink, Grace Y. Gombolay, Kathleen M. Cardinale, Diego Real de Asua, Shahid Zaman, Stephanie L. Santoro, Sammer M. Marzouk, Mellad Khoshnood, Benjamin N. Vogel, Runi Tanna, Dania Pagarkar, Sofia Dhanani, Maria del Carmen Ortega, Rebecca Partridge, Maria A. Stanley, Jessica S. Sanders, Alison Christy, Elise M. Sannar, Ruth Brown, Andrew A. McCormick, Heather Van Mater, Cathy Franklin, Gordon Worley, Eileen A. Quinn, George T. Capone, Brian Chicoine, Brian G. Skotko, Michael S. Rafii

**Affiliations:** ^1^Department of Pediatrics, Children's Hospital Los Angeles, Los Angeles, CA, United States; ^2^Department of Neurology, Keck School of Medicine at USC, Los Angeles, CA, United States; ^3^Department of Psychiatry, University of Colorado School of Medicine, Denver, CO, United States; ^4^Department of Neurology, University of Colorado Anschutz Medical Campus, Aurora, CO, United States; ^5^Department of Pediatrics, University of Pittsburgh School of Medicine, Pittsburgh, PA, United States; ^6^Department of Pediatrics, Division of Neurology, Emory University and Children's Healthcare of Atlanta, Atlanta, GA, United States; ^7^Department of Neurology, Yale University School of Medicine, New Haven, CT, United States; ^8^Adult Down Syndrome Outpatient Clinic, Department of Internal Medicine, Fundación de Investigación Biomédica, Hospital Universitario de La Princesa, Madrid, Spain; ^9^Cambridge Intellectual & Developmental Disabilities Research Group, Department of Psychiatry, University of Cambridge, Cambridge, United Kingdom; ^10^Down Syndrome Program, Division of Medical Genetics and Metabolism, Department of Pediatrics, Massachusetts General Hospital, Boston, MA, United States; ^11^Department of Psychiatry, Clinica Universidad de Navarra, Madrid, Spain; ^12^Virginia Mason Health System, Issaquah, WA, United States; ^13^Department of Pediatrics, University of Wisconsin School of Medicine and Public Health, Madison, WI, United States; ^14^Sie Center for Down Syndrome at the University of Colorado, Aurora, CO, United States; ^15^Providence Health System, Portland, OR, United States; ^16^Division of Psychiatry and Behavioral Sciences, Children's Hospital Colorado, Aurora, CO, United States; ^17^Department of Psychology, Virginia Commonwealth University, Richmond, VA, United States; ^18^Division of Rheumatology, Department of Pediatrics, Duke University, Durham, NC, United States; ^19^Queensland Center for Intellectual and Developmental Disability, Mater Research Institute, The University of Queensland, South Brisbane, QLD, Australia; ^20^Division of Pediatric Neurology and Developmental Medicine, Department of Pediatrics, Duke University School of Medicine, Durham, NC, United States; ^21^Department of Pediatrics, University of Toledo College of Medicine and Life Sciences, Toledo, OH, United States; ^22^Department of Pediatrics, Kennedy Krieger Institute, Baltimore, MD, United States; ^23^Department of Pediatrics, Johns Hopkins School of Medicine, Baltimore, MD, United States; ^24^Advocate Medical Group Adult Down Syndrome Center, Park Ridge, IL, United States; ^25^Department of Pediatrics, Harvard Medical School, Boston, MA, United States; ^26^Department of Neurology, Alzheimer's Therapeutic Research Institute (ATRI), Keck School of Medicine at the University of Southern California, San Diego, CA, United States

**Keywords:** Down syndrome, regression, criteria & indicators, consensus, encephalopathy

## Abstract

**Objective:**

To develop standardization for nomenclature, diagnostic work up and diagnostic criteria for cases of neurocognitive regression in Down syndrome.

**Background:**

There are no consensus criteria for the evaluation or diagnosis of neurocognitive regression in persons with Down syndrome. As such, previously published data on this condition is relegated to smaller case series with heterogenous data sets. Lack of standardized assessment tools has slowed research in this clinical area.

**Methods:**

The authors performed a two-round traditional Delphi method survey of an international group of clinicians with experience in treating Down syndrome to develop a standardized approach to clinical care and research in this area. Thirty-eight potential panelists who had either previously published on neurocognitive regression in Down syndrome or were involved in national or international working groups on this condition were invited to participate. In total, 27 panelists (71%) represented nine medical specialties and six different countries reached agreement on preliminary standards in this disease area. Moderators developed a proposed nomenclature, diagnostic work up and diagnostic criteria based on previously published reports of regression in persons with Down syndrome.

**Results:**

During the first round of survey, agreement on nomenclature for the condition was reached with 78% of panelists agreeing to use the term Down Syndrome Regression Disorder (DSRD). Agreement on diagnostic work up and diagnostic criteria was not reach on the first round due to low agreement amongst panelists with regards to the need for neurodiagnostic testing. Following incorporation of panelist feedback, diagnostic criteria were agreed upon (96% agreement on neuroimaging, 100% agreement on bloodwork, 88% agreement on lumbar puncture, 100% agreement on urine studies, and 96% agreement on “other” studies) as were diagnostic criteria (96% agreement).

**Conclusions:**

The authors present international consensus agreement on the nomenclature, diagnostic work up, and diagnostic criteria for DSRD, providing an initial practical framework that can advance both research and clinical practices for this condition.

## Introduction

Down syndrome (DS) is the most common cause of genetic intellectual disability worldwide and occurs in 1 in 800–1,000 live births, with more than 214,000 people with DS living in the United States and 417,000 in Europe (1, 2). Neurologic and psychiatric conditions in this population are well established although an increasing number of reports of a subacute neurocognitive regression of unclear etiology have been published over the last decade (3–7). This condition has been referred to as Down syndrome disintegrative disorder (DSDD), Down syndrome regression disorder (DSRD) and unexplained regression in Down syndrome (URDS) (6–8). Symptoms of this condition include subacute loss or deterioration of previously acquired developmental skills in the domains of language, communication, cognition, executive function, behavioral, and adaptive skills (1, 5–7, 9–12). Neuropsychiatric symptoms including catatonia, agitation, hallucination, and depersonalization have also been reported (3–8). This condition is frequently severe and impacts the quality of life of both persons with DS and their caregivers, highlighting the need for expedited research in this area (6–9).

Very little is known about this condition with the majority of studies being limited to smaller data sets and case series. Study to date has compared clinical features between persons with DS and regression and persons with DS without regression, supporting a 28-item clinical definition (8), however, a lack of consistent diagnostic approaches and criteria have hindered research in this area, yielding small studies that are heterogeneous and lack broad generalizability. Limiting dedicated study of this condition are (1) consistent nomenclature, (2) guidance on evaluation of individuals with suspicion for neurocognitive regression in persons with DS and (3) definitive clinical diagnostic criteria for the condition. Dedicated advancement of research in this field has been identified as high need by clinicians and researchers in the field, highlighting the need for tools, such as standardized approaches to assessment, to move forward in characterizing, diagnosing, and treating individuals with this condition (13).

This study sought to survey individuals with expertise in neurocognitive regression in persons with DS using the traditional Delphi method in order to create a multi-disciplinary expert guided consensus on the diagnostic work up and criteria for regression in persons with DS. This study may be of benefit for both future research in regression in DS and for clinical guidance in the diagnosis of this condition.

## Methods

### Choice of Assessment Methodology

The Delphi method is an established, multi-stage, process for developing consensus using at least two rounds of anonymous surveys (14). This methodology is often utilized when there is a lack of scientific evidence to make recommendations and is frequently utilized in rare and ultra-rare conditions. The advantages of the method include the ability to involve a large group of international participants, low cost, anonymity, and reducing the likelihood of dominance by certain participants. Given that in-person workgroups were not realistic during the COVID19 pandemic, this research methodology was prioritized. For this study a traditional two-step Delphi method was utilized.

### Moderators Panel

The moderator group was comprised of one neurologist, one developmental pediatrician, one clinical psychologist, and one psychiatrist with expertise in the diagnosis and treatment of DSRD. This group had previously worked together on multi-center collaborations in the field of neurocognitive disorders in DS. This group was previously formed and no selection of moderators was made. All panelists were blinded to members of the moderator panel with the exception of the first author who was responsible for recruitment of panelists *via* e-mail.

### Panelist Selection

Experts in DSRD were defined as individuals who had evaluated >10 individuals with DSRD, had published a manuscript, had presented at a national or international conference or who worked on or led a national or international consortium on this disorder. Recruitment to the panel was made through e-mail invitation to physician or psychologist members of the regression sub-committee of the Down Syndrome Medical Interest Group (DSMIG) which is a multi-disciplinary team of medical professionals treating and researching individuals with DSRD. In addition, any corresponding author of a manuscript of DSRD (including when URDS, DSRD, or similar nomenclature were utilized) in the last 20 years was also offered an invitation to join the panel. In addition, to these two inclusion criteria, other authors on published reports who were not the corresponding author were also offered involvement in participating in the study. Panelists were excluded if they were not physicians (defined as medical doctor or doctor of osteopathy) or psychologists (defined as PhD in a clinical field). Given the uncertain etiology (and potential for multiple etiologies) of DSRD, a minimum of two individuals with medical expertise and training in psychology, psychiatry, neurology, and primary care was set to ensure appropriately broad views on DSRD as well as broad diagnostic approaches to the condition. If no response was received, invitations were emailed on a second occasion 2 weeks after the initial invitation. Moderators did not participate in surveys. There was no pre-defined limit to the maximum number of panel participants. Invitations for participation were made on November 29^th^, 2021, and, when necessary, a second invitation was sent on December 13^th^, 2021.

### Panel Membership

In total, 38 individuals were invited to participate on the panel with 27 (71%) participants completing the first survey. To encourage participation, those who did not respond were sent a maximum of two additional e-mail messages encouraging participation. Demographic information on the panelists from the first round is provided in Table 1. Subsequently, 25 participants completed the secondary survey, all of whom had previously completed the first survey as well. Two individuals who completed the first survey elected to not participate in the second round of the survey and thus did not receive additional communication from the study group. All first and second round surveys were completed in full. The median time from e-mail invitation to survey completion was 32 h (IQR: 17–49).

**Table 1:** Demographics of expert panel (*n* = 27).

	***n*/*N* (%)**
**Region**	
United States	22 (81%)
Europe	3 (11%)
Asia/Australia	2 (7%)
**Age**	
20–30 years	2 (7%)
31–40 years	7 (26%)
41–50 years	12 (44%)
51–60 years	2 (7%)
61+ years	4 (15%)
**Sex**	
Female	10 (38%)
Male	16 (62%)
**Race**	
Caucasian/White	22 (81%)
Asian	4 (15%)
Black/African American	0
Native American	0
Mixed Race/Other	1 (4%)
**Ethnicity**	
Hispanic/Latino	1 (4%)
Not Hispanic/Latino	26 (96%)
**Clinical field of practice**	
*Neurology*	10 (37%)
Pediatric neurology^a^	6 (22%)
Pediatrics	4 (15%)
Psychiatry	3 (11%)
Psychology (PhD)	5 (11%)
Genetics	2 (7%)
Internal medicine/family medicine	2 (7%)
Immunology	2 (7%)
Developmental and behavioral pediatrics	1 (4%)
Other	0
**Years of clinical experience**	
0–5	7 (26%)
6–10	10 (37%)
11–15	3 (11%)
16–20	3 (11%)
21–25	1 (4%)
26–30	1 (4%)
30+	2 (7%)
**Ages evaluated**	
Adults only	3 (12%)
Children only	7 (28%)
Both	15 (60%)
**Cases of regression evaluated**	
0–20	14 (52%)
21–50	5 (19%)
51–100	4 (15%)
100–200	0
200+	1 (4%)

a *Pediatric neurologists could also identify as neurologists or pediatricians in this study*.

### Definitions of Agreement and Consensus

Agreement was reported as a percentage and defined as the percentage of positive responses (agree or strongly agree) divided by the total number of responses. Responses were not weighted, and panelist seniority, specialty, or total cases evaluated were not considered. Consensus was defined *a priori* as >75% of responses meeting either agree or strongly agree, similar to other studies in adjacent rare neurologic disorders (11, 15, 16).

### Development of Initial Recommendations for Panel

Nomenclature options were developed by reviewing literature of published cases of developmental regression in DS. Diagnostic medical evaluation recommendations were made by reviewing previously published and institutional data on cases of regression (4–9), review of high prevalence disorders that are treatable in individuals with DS (17–22), and neurodiagnostic studies commonly utilized in both autoimmune encephalitis (23, 24) and unexplained developmental regression (6–8). The latter two components were reviewed and utilized as a framework for the work up and evaluation of patients who have subacute neurocognitive deterioration although the authors believe that regression in persons with DS is ultimately a dissimilar process. Studies were divided into recommendations for all patients or only “if clinically indicated” leaving judgement to the physician or clinical team in the latter category. Clinical diagnostic criteria were developed by reviewing existing formats for the diagnosis of autoimmune encephalitis and encephalopathy (23, 24), analysis of existing published phenotypic data in regression in persons with DS and review of previously published and institutional data in cases of established regression (3–8, 10). In addition, data from studies regarding co-morbid non-regression related causes of regression in persons with DS (e.g., autism spectrum disorders) were also reviewed in order to minimize overlapping diagnostic criteria and differentiate cases (17–22, 25).

The moderator generated diagnostic criteria were evaluated against an existing DSRD data set prior to release to the panel (26). This was performed as a quality control measure to ensure that panelists would receive as high quality a proposal as possible. This process involved application of the moderator proposed diagnostic criteria and applying it to confirmed cases of DSRD that were previously published. The criteria for “possible” DSRD yielded a 100% sensitivity and 79% specificity. Similarly, “probable” DSRD criteria yielded a 94% sensitivity and a 93% specificity. Following confirmation that proposed criteria were reasonable for the identification of DSRD by the moderator panel, the decision to engage panelists was made.

### Survey Administration and Rounds

Surveys were distributed to panelists by e-mail invitation. Interested panelists then followed a hyperlink to the REDCap (version 12.1.1, Vanderbilt University) survey home page where responses were entered (12). The original proposal was drafted by the moderator panel based on clinical experience in the evaluation of individuals with DSRD and previously reported data.

The first round of the Delphi process asked participants to evaluate moderator panel proposed nomenclature, diagnostic work up, and diagnostic criteria. Participants were offered multiple selection options (strongly agree, agree, disagree, strongly disagree), although only one response could be recorded. All questions, regardless of the response provided, contained open-ended sections seeking additions to or deletions from the original suggestions. Panelists had 2 weeks to complete the initial survey. Following the end of round one, moderators made the suggested revisions to the corresponding proposal.

The criteria for integrating feedback between the first and second round of the survey was uniform. In the first round, an item was carried forward or edited if it was identified by at least two panel members. The rationale for these criteria was that an item endorsed by only one panel member or attracting <5% of the total scoring variance, was not clinically important in the view of the expert panel as a whole. These criteria were intended to keep in play any items that the group might ultimately consider important and to exclude items unlikely to be considered important in any subsequent round.

A second round followed and asked panelists to provide input on diagnostic work up and diagnostic criteria. This was again performed using a REDCaps survey. Only individuals who completed the first round of the survey were invited to participate in the second round. As consensus had been reached on nomenclature during round one of the survey, this was not included for additional evaluation. Panelists were offered selection options (agree or disagree only) but open-ended responses were not provided after each section. Each section was offered as a combined field (e.g., criteria 1–4 listed jointly). Reponses of panelists (anonymized) were provided from the first round for review. A final comments section was provided to all panelists for any additional input. Panelists had 2 weeks to complete the second survey and e-mail reminders were administered on two occasions to optimize full panelist retention. All changes made between the first and second surveys were clearly listed at the bottom of each section of the second survey for rapid review by panelists.

After completion of the second survey, consensus was determined to have been obtained as noted above for all components of the proposals. Final edits were made by the moderator panel and are reviewed below.

### Statistical Analysis

Demographic data was collected and reported as a percent of the total participants in round one of the surveys. Responses to survey questions in both rounds one and two were reported as a frequency (total responses of one type divided by the number of all responses). When determining consensus, responses were clustered as “strongly agree” and “agree” or “strongly disagree” and “disagree.” Odds ratios were calculated to determine if panelist demographics influenced the likelihood of particular response although the cohort was too small for formal regression analysis. Specificity and sensitivity were calculated on the inception cohort used to generate recommendations after consensus criteria were developed.

## Results

**Figure 1:**
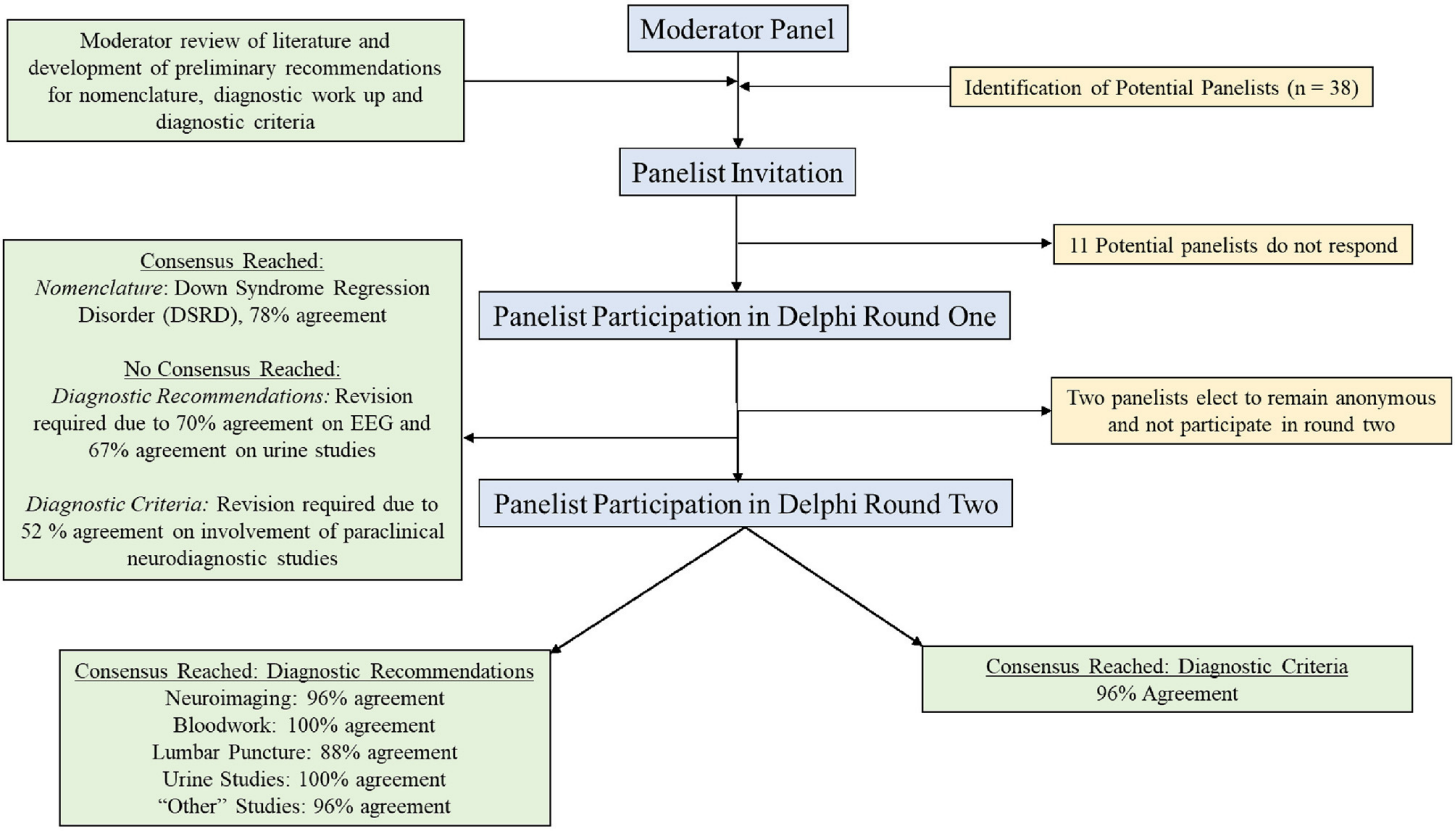
Flow diagram for panelist selection and Delphi method assessment.

A flow diagram of the selection process and voting process is displayed as Figure 1.

### Nomenclature

Consensus for nomenclature for this condition was Down Syndrome Regression Disorder (DSRD), with 78% (*n* = 21) of all votes in favor of this terminology (Table 2). Consensus was reached on the first survey. Down syndrome disintegrative disorder received 19% of vote (*n* = 5) and unexplained regression in Down syndrome (URDS) received 4% (*n* = 1). Open responses from panelists were appreciated in two responses (8%) and included “URDS indicates that there is no etiology” and “DSDD has negative connotations as a permanent/static condition”.

**Table 2:** Expert panel responses.

	**Initial responses**				**Consensus^**1**^ reached round 1?**		**Subsequent responses (grouped)**		**Consensus^**1**^ reached round 2?**
**Nomenclature**										
*DSRD*	21 (78%)				Yes					
*DSDD*	5 (19%)								
*URDS*	1 (4%)								
*Other*	0								
	***SA***	***A***	***D***	***SD***	**–**		***A***	***D***	***–***
**Diagnostic work up**						**Diagnostic work up**			
Neuroimaging (All)	15	7	4	1	Yes, 81%	Neuroimagin*g*	24	1	Yes, 96%
Neuroimaging (ICI)	8	16	1	2	Yes, 89%				
Blood work (All)	18	8	1	0	Yes, 96%	Blood work	25	0	Yes, 100%
Blood work (ICI)	9	12	5	1	Yes, 78%				
LP (All)	9	12	5	1	Yes, 78%	LP	22	3	Yes, 88%
LP (ICI)	6	15	5	1	Yes, 78%				
EEG (All)	10	12	5	0	Yes, 81%	EEG	24	1	Yes, 96%
EEG (ICI)	7	12	8	0	No, 70%				
Urine (All)	5	13	7	2	No, 67%	Urine	25	0	Yes, 100%
Urine (ICI)	4	16	5	2	No, 74%				
Other (All)	3	18	4	2	Yes, 78%	Other	24	1	Yes, 96%
Other (ICI)	11	13	3	0	Yes, 89%				
**Diagnostic criteria**									
Category 1	15	11	1	0	Yes, 96%				
Category 2	20	6	1	0	Yes, 96%	**Diagnostic criteria**	24	1	Yes, 96%
Category 3	8	6	12	1	No, 52%				
Category 4	18	7	2	0	Yes, 93%				

*A, agree; D, disagree; DSDD, Down syndrome disintegrative disorder; DSRD, Down syndrome regression disorder; ICI, if clinically indicated; SA, strongly agree; SD, strongly disagree; URDS, unexplained regression in Down syndrome*.

*Consensus defined as strongly agree and agree responses totaling >75% of all responses*.

### Diagnostic Work Up

Round one survey responses regarding diagnostic evaluations are displayed in Table 2. Across 12 total fields, consensus agreement during round one was present in nine fields (75%). Fields lacking *a priori* consensus were EEG testing (if clinically indicated), urine testing (all patients) and urine testing (if clinically indicated) receiving 70% (*n* = 19), 67% (*n* = 18), and 74% (*n* = 20) agreement, respectively. All fields garnered panelist feedback suggesting revision with six fields (50%) meeting criteria for revision as noted above. Panelists with >15 years of clinical experience (*n* = 7) were more likely to vote “disagree” or “strongly disagree” on all diagnostic studies except neuroimaging and bloodwork (OR: 2.47, 95%CI: 1.28–4.79, *p* = 0.01). Other demographic factors did not impact responses and were evenly distributed. Selected open responses regarding each diagnostic study were broad and are contained in Appendix 1.

Responses to modified (second round) diagnostic work up recommendations presented in the second stage of the survey are also presented in Table 2. As feedback necessitated review of all fields, each was included again for review by the expert panel although were grouped in one unit given the multiple suggestions to do so by panelists. Agreement on the second round of survey was 96% (*n* = 24) for neuroimaging, 100% (*n* = 25) for bloodwork, 88% (*n* = 22) for lumbar puncture, 100% (*n* = 25) for urine studies, and 96% (*n* = 24) for “other” studies. Thus, all fields met pre-defined consensus cut-offs of >75% agreement during the second round of surveys. No additional comments were made by panelists during round two regarding diagnostic recommendations.

### Criteria for DSRD

Initial responses to the proposed criteria for DSRD are included in Table 2. A high degree of agreement was reached for nearly all fields with the exception of criteria three (paraclinical neurodiagnostic studies) which reached 52% (*n* = 14) agreement. All other fields reached a minimum of 93% agreement. Disagreement on criteria three was notably driven by non-neurologists who voted either disagree or strongly disagree on this metric (10/13, 77% were non-neurologists, *p* = 0.02, 95%CI: 1.47–47.22). Other demographic factors did not impact responses and were evenly distributed. Selected open response feedback regarding diagnostic criteria is contained in Appendix 1.

In round two, the criteria were restricted to excluded paraclinical neurodiagnostic studies. Agreement was subsequently 96% (*n* = 24; Table 1). No additional feedback was offered during this second round of study.

### Validation of Diagnostic Criteria

Consensus diagnostic criteria were re-applied to the inception cohort that was originally used to generate the proposed diagnostic criteria referenced in the methods section (26). Application of the subsequent diagnostic criteria improved specificity to 100% for “possible” DSRD and 98% for “probable” DSRD. Similarly, sensitivity of these criteria improved to 84% for “possible” DSRD and 92% for “probable” DSRD.

## Discussion

DSRD is a poorly defined neurocognitive disorder that can have devastating effects on persons with DS and their families (6, 7, 9, 10). Individuals with DS are presenting with symptoms of DSRD to clinics both nationally and internationally, but a lack of clear guidance on diagnosis and work up has impeded standardized recognition and care of these individuals. Further, variation in terminology has made it difficult to compare cases across settings. This not only impacts more immediate options for diagnosis and treatment but also creates challenges when attempting to research understanding of the etiologies of this condition and has been identified as a high need area in DS-related research (13).

Utilizing the traditional Delphi method (14), this study collected feedback from an international group of clinicians with expertise in the care of individuals with both DS and DSRD. These agreed upon recommendations for diagnostic work up and clinical diagnosis are displayed in Tables 3, 4, respectively. The data-driven approach to this survey allowed for rapid consensus amongst an international group of experts across several medical disciplines.

**Table 3:** Consensus recommendations for the work up of regression in Down syndrome.

	**All patients**	**As clinically indicated**
**Diagnostic imaging**	Brain MRI with and without gadolinium contrast on a 3T scanner	MRI spine with and without contrast PET/SPECT imaging MR angiography of the head and neck MR spectroscopy
**Blood tests**	Ammonia CBC w/differential CMP ESR CRP Lipid panel HbA1c B12 level Vitamin D 25-OH level TSH w/reflex T4 TPO antibodies Anti-thyroglobulin antibodies Anti-thyroid stimulating hormone receptor ANA Celiac serology or panel Cell-based autoimmune encephalitis panel	Infectious testing^a^^,^^b^ dsDNA Complement 3 and 4 Immunoglobulin levels Cytokine panel Celiac panel ASO Anti-DNAse B Vitamin B1 level Methylmalonic acid Vitamin B6 level Homocysteine level Iron level, TIBC, and Iron Saturation Selenium level Heavy metal screen (lead, manganese, mercury, zinc, nickel, thallium) Myelin oligodendrocyte glycoprotein (MOG) antibodies (if not covered in cell-based panel) Lactate Pyruvate Advanced biochemical profiling (neurometabolic disorder evaluation) Fragile X testing Chromosomal Microarray Whole exome sequencing
**Urine tests**	n/a	Urinalysis with reflex culture Urine toxicology Total porphyrin and porphobilinogen Organic acids Acylglycines Glycosaminoglycans Oligosaccharides Sialic acid
**Lumbar puncture**	Cell count with differential Total protein Glucose Gram stain and culture IgG index Oligoclonal bands Cell-based autoimmune encephalitis panel	Infectious testing^a^^,^^b^ Opening Pressure Neopterin Angiotensin converting enzyme (ACE) Lactate Pyruvate CSF amino acids Alpha aminoadipic semialdehyde Folate receptor antibody assay 5-Methyltetrahydrofolate Tetrahydrobiopterin Neurotransmitter metabolites (homovanillic acid, 3-*O*-methyl-dopa, and 5-hydroxyindole acetic acid) Pyridoxal 5′-phosphate Sialic acid Succinyladenosine Sepiapterin and dihydrobiopterin Amyloid-beta 42/40 Phosphorylated tau
**Electroencephalogram**	Routine (60 min) EEG	Prolonged EEG (4–6 h) Overnight EEG (24+ h)
**Other testing**	n/a	Polysomnogram (OSA evaluation) Audiogram (hearing evaluation) Neurocognitive assessment

a *Potential bacterial/protozoal infectious testing: Borrelia burgdorferi, HIV, Listeria monocytogenes, Mycoplasma pneumoniae, Mycobacterium tuberculosis, Treponema pallidum*.

b *Potential viral infectious testing: adenovirus, enterovirus, Epstein-Barr virus, herpes simplex virus 1 and 2, human herpes virus 6 and 7, influenza virus A and B, John Cunningham virus, measles, rabies, varicella zoster, west Nile virus and other region-dependent viral testing*.

**Table 4:** Consensus recommendations for the diagnosis of Down syndrome regression disorder.

**Category**	**Criteria**	**Possible DSRD**	**Probable DSRD**
Symptom onset	Onset of new neurologic, psychiatric, or mixed symptoms over a period of <12 weeks in previously health individual with Down syndrome	Yes	Yes
Clinical evidence of neurologic dysfunction	1. Altered mental status or behavioral dysregulation - Anorexia/decreased oral intake or hyperphagia - Confusion/disorientation - Inappropriate laughter - Encephalopathy 2. Cognitive decline - Apathy - Abulia and/or avolition - Acute memory impairment (including new difficulty with recall) 3. Developmental regression with or without new autistic features - Social withdrawal - Loss of previously developmental acquired milestones - Inability to perform activities of daily living - Stereotypy - Rigidity around routine changes - Decreased eye contact 4. New focal neurologic deficits on examination and/or seizure 5. Insomnia or circadian rhythm disruption 6. Language deficits - Expressive and/or receptive aphasia - Global aphasia (mutism) - Whispered speech 7. Movement disorder (excluding tics)^*^ - Catatonia - Bradykinesia - Freezing - Gait disturbance 8. Psychiatric symptoms -Anxiety -Delusions or hallucinations - Derealization/depersonalization - Obsessive compulsive tendencies -Aggression/agitation	>3 symptom clusters present	>6 symptom clusters present
Exclusion of other etiologies	Reasonable exclusion of alternative causes of regression including other systemic and central nervous system disorders. Other primary psychiatric disorders are also considered exclusionary	Yes	Yes

* *Must be included as one of the symptom clusters for possible or probable diagnosis*.

In general, agreement was high with nearly three quarters of responses on the first survey meeting *a priori* consensus definition. This early agreement was presumably driven by the development of proposed diagnostic work up and diagnostic criteria from real-world data considering the heterogeneity of the panelist team. Panelists with more significant clinical experience (>15 years) were more likely to disagree or strongly disagree with diagnostic study recommendations. The reasons for this are unclear although informal *post hoc* interview of veteran clinicians (*n* = 5) revealed a consistent theme of preferring to treat patients clinically as opposed to relying on adjunct diagnostic algorithms. In addition, three clinicians in this group (60%) reported that they felt uncomfortable ordering tests that they would not feel comfortable evaluating independently (e.g., interpretation of an EEG). Exclusion of neurodiagnostic studies (criteria three) from the final diagnostic criteria was driven largely by non-neurologists. As neuroimaging, EEG, and lumbar puncture are all frequently in the purview of the neurologist for both ordering and interpretation, this result was not surprising although it highlights the importance of utilizing the Delphi method to avoid skew of recommendations based on demographics of panelists.

The proposed diagnostic workup rests upon the premise that the main differential diagnoses for this condition are either neurologic or psychiatric in origin as the basis for standardization of testing is designed to rule out medical explanations for symptoms. This is an important consideration for clinicians utilizing these consensus recommendations as we currently lack sensitive and specific biomarkers for the diagnosis of DSRD. The hypothesis for neurologic and/or psychiatric origins in DSRD is based upon data reporting therapeutic benefit from both immunomodulatory therapies (e.g., steroids, IVIg, and rituximab) as well as therapies for catatonia (e.g., lorazepam) and other psychiatric diseases (e.g., antidepressants, antipsychotics, and electroconvulsive therapy) (3–5, 26, 27). The panelists in this study were largely of neurology, psychiatry or psychology backgrounds (18/27, 67%), which may have influenced the rationalization for testing recommendations although there were no differences in likelihood of rating particular testing more or less frequently based on field of practice.

Flexibility regarding “possible” and “probable” diagnoses of DSRD were well received by panelists. Although these designations are much more unique to the field of neurology, and specifically autoimmune encephalitis (23, 24), panelists agreed that clinical phenotypes of DSRD may vary over time and may be more or less concerning as their clinical course unfolds. A caveat to these designations brought up by panelists was that treated patients may move from “probable” to “possible” criteria if they clinically improve, and thus, should be operationally used during initial evaluations and not after treatment is initiated (rather, referring to a patient as having only DSRD). Panelists favored not utilizing characterization of disease severity due to the highly subjective nature of this and the need to collect additional natural history data before accurate characterization could be performed.

The impact of this study lies in the ability to provide a framework for future investigations into DSRD. By unifying a clinical approach to the work up and diagnosis of this condition, more consistent retrospective and prospective studies can be performed on this unique, and poorly described condition. Certain factors were purposely excluded from the proposed diagnostic criteria such as age ranges and phenotyping of severity as panelists felt more data was needed before incorporating into these recommendations. The authors acknowledge that as further data is collected and published, revision of both the proposed diagnostic work up and diagnostic criteria will be necessary. For this reason, the authors have proposed a reconvening (with additional panelist recruitment) in a period of 5 years (2027). It is the hope of the authors that significantly more data will be available for review at that time, necessitating review and revision of the proposed criteria in this study.

This study is not without limitations. This study used the Delphi method for capture of agreement on a variety of measures. While the Delphi method is considered a preferred consensus measure, limitations of this method include the use of arbitrary cut-offs for consensus and potential for influence of moderators. To mitigate these factors, the authors utilized an *a priori* cutoff of 75% consensus which is utilized in similar studies in rare neurologic disease (11, 15, 16). Although consensus diagnostic work up recommendations were rendered, these studies are designed to rule out other conditions that could potentially mimic DSRD as opposed to “ruling in” the condition as there are a lack of definitive biomarkers of the disease available at this time. This is an important consideration for clinicians utilizing these recommendations and highlights the importance of having a multi-disciplinary team evaluating cases of DSRD. Regarding the influence of moderators, the moderators did not directly communicate with panelists nor were their responses collected for integration into the consensus criteria. DSRD is thought to be a rare condition which is why consensus criteria were proposed originally and this is noted in the generally low number of patients evaluated by most members of the expert panel. Bias in the selection of panelists using the Delphi method is possible. To minimize panel selection bias the authors solicited any panelists who had published in the field previously (as a corresponding author) and also solicited panelists from multiple different medical specialties. There was a higher rate of neurology representation in this study although this was felt to be more reflective of the identification of unique neurologic features in this condition over the past decade which has reflected a paradigm shift away from viewing DSRD as strictly psychiatric in nature (3, 6–10). In addition, the use of non-weighted determination of consensus was made to avoid skewing data toward larger academic medical centers and clinics. While panelists represented an international group, there was a significant skew toward centers from the United States which limits some of the generalizability regarding the feasibility of the diagnostic work up proposed. This geographic limitation is also likely reflective of the fact that individuals involved with DSMIG or who had previously published on DSRD were mostly from the United States or United Kingdom. Thus, it is possible that clinicians with experience treating DSRD were not included in this study. Finally, validation of these criteria could only be performed on the inception cohort noted in the methods section as insufficient data was available in nearly all literature reviewed. While the sensitivity and specificity of diagnosis of DSRD both improved with application of the proposed criteria, the authors intend to perform prospective validation of these criteria.

In summary, this study describes the first systematic consensus process for DSRD from an international group of experts in DS and regression. These findings will hopefully be used as a launch point for further research investigations into this growing field although will require monitoring and revision as more information is learned.

## Data Availability Statement

The original contributions presented in the study are included in the article/Supplementary Material, further inquiries can be directed to the corresponding authors.

## Author Contributions

JDS, LP, MR, and EQ conceptualized the study, collected data, analyzed data, and synthesized data. These authors drafted the manuscript and edited for intellectual content. RK, RF, GG, KC, DA, SZ, SS, MK, ES, MO, RP, MS, JSS, AC, RB, AM, HM, GW, GC, and BC provided input in study design, reviewed data, and edited the manuscript for intellectual content. SM and BV collected and reviewed data and edited the manuscript for intellectual content. MK, RT, DP, and SD analyzed data, performed quality control of data and edited the manuscript for intellectual content. All authors contributed to the article and approved the submitted version.

## Conflict of Interest

JDS receives research support from the National Institutes of Health, the Race to Erase MS Foundation, and the Thrasher Society. He also serves as a consultant to UCB on myelin oligodendrocyte glycoprotein antibody spectrum disorders. GG receives part-time salary support from the Centers for Disease Control and Prevention for acute flaccid myelitis disease surveillance. DA is supported by the Fondo de Investigaciones Sanitarias (FIS grant PI19/00634, from the Ministerio de Economía y Competitividad (Instituto de Salud Carlos III) and co-funded by The European Regional Development Fund (ERDF) “A way to make Europe” and the. Fondation Jérôme Lejeune (grant no. 2021a-2069). The Adult Down Syndrome Outpatient unit at Hospital Universitario de La Princesa is grateful to Licenciado don Jesús Coronado Hinojosa for his financial support of the research endeavors of our unit. BS occasionally consults on the topic of Down syndrome through Gerson Lehrman Group. He receives remuneration from Down syndrome nonprofit organizations for speaking engagements and associated travel expenses. BS receives annual royalties from Woodbine House, Inc., for the publication of his book, Fasten Your Seatbelt: A Crash Course on Down Syndrome for Brothers and Sisters. Within the past 2 years, he has received research funding from F. Hoffmann-La Roche, Inc., and LuMind Research Down Syndrome Foundation to conduct clinical trials for people with Down syndrome. BS is occasionally asked to serve as an expert witness for legal cases where Down syndrome is discussed. He serves in a nonpaid capacity on the Honorary Board of Directors for the Massachusetts Down Syndrome Congress and the Professional Advisory Committee for the National Center for Prenatal and Postnatal Down Syndrome Resources. BS has a sister with Down syndrome. The remaining authors declare that the research was conducted in the absence of any commercial or financial relationships that could be construed as a potential conflict of interest.

## Publisher's Note

All claims expressed in this article are solely those of the authors and do not necessarily represent those of their affiliated organizations, or those of the publisher, the editors and the reviewers. Any product that may be evaluated in this article, or claim that may be made by its manufacturer, is not guaranteed or endorsed by the publisher.
