# Supplementary material for: Assessment and Diagnosis of Down Syndrome Regression Disorder: International Expert Consensus
Source: Front Neurol. 2022 Jul 15;13:940175. doi: 10.3389/fneur.2022.940175 (PMC9335003; doi:10.3389/fneur.2022.940175)
Supplement: Supplementary file 1 [file Data_Sheet_1.DOCX]

**Appendix 1:** Selected open responses during the first phase of Delphi survey

Regarding Diagnostic Studies:

1. *Neuroimaging*
   1. “If diagnosis is strongly suspected and neurological physical exam is normal, both MRI or CT might be appropriate options, and even some cases could be handled without imaging”
   2. “magnetic resonance spectroscopy”
   3. “Unless there is baseline hypertension or features of focal abnormalities on exam, evaluation with vessel imaging and spine imaging seems low yield.”
   4. “In patients with prior MRI, it would be useful to compare findings”
   5. “Interpretation of normal should be considered for persons with Down syndrome… for example, smaller cerebellums can be observed in this population with regularity but may not be seen in age matched neurotypical individuals”
2. *Bloodwork*
   1. “Lipid panel is irrelevant, ammonia only required if prior history of hepatic damage and encephalitis panel not universally available”
   2. “Would be okay with not obtaining an ammonia level on all patients unless there is Depakote use.”
   3. “autoimmune panel based on clinical suspicion”
   4. “Agree with doing these as clinically indicated. Concerned that inclusion of some of these will lead to unnecessary testing and lab results which may not have value of clinical significance. For example, so many individuals could have positive ASO and anti-DNAse B without this helping understand etiology. also, why not add ceruloplasmin?”
   5. “Uncertain what yield genetic testing would provide, though of course the presence of one genetic anomaly does not preclude the presence of other genetic anomalies.”
   6. “In my opinion, blood work has been the lowest yield of all testing”
3. *Lumbar Puncture*
   1. “Serum glucose, 5-MTHFR, tetrahydrobiopterin, Neurotransmitters”
   2. “feel less strongly about neopterin although I do understand the link in inflammatory brain disorders”
   3. “I would include CSF neurotransmitters and amino acids in necessary for all patients”
   4. “I agree this is ideal, but not always logistically possible.”
   5. “haven't done, to date, given the challenges of obtaining LP for actively catatonic patients”
   6. “Ideally, this should be performed under sedation with MRI but logistically challenging”
4. *EEG*
   1. “Consider 24 hour or at least EEG spanning awake and asleep”
   2. “I would only recommend an EEG if seizures or seizure-like symptoms”
   3. “I think a prolonged study to ensure the capturing of sleep would increase the overall yield of the study.”
   4. “I haven't seen sufficient evidence to indicate this is needed for ALL patients.”
   5. “EEG must be compared to prior studies if available as these can be abnormal in many healthy persons with Down syndrome”
5. *Urine Studies*
   1. “Urine organic acids should be necessary”
   2. “Low yield”
   3. “Hard to obtain in most patients outside of the inpatient setting”
6. *Other Studies*
   1. “Neurocognitive assessment would be ideal, preferably before and after therapy”
   2. “May want to consider polysomnogram on all patients since studies show caregivers are not good reporters of symptoms of OSA”
   3. “PSG, audiogram should be strongly considered if not recently completed”
   4. “Whole exome”

Regarding Diagnostic Criteria:

1. Category 1:
   1. “This is consistent with diagnostic criteria for auto-immune encephalitis, and I think provides a good framework of when to suspect the disorder. It remains broad enough to catch more subtle cases.”
   2. “Less than 12 weeks is not enough time in my opinion, as other diagnoses could be mimicking - i.e. I need more information”
   3. “The timing of this criteria seems appropriate. I find that almost no patients are being evaluated that quickly from the onset of symptoms so there is time to make the appropriate diagnosis”
2. Category 2:
   1. “I would describe focal Neuro exam findings of concern; movement disorder would fit this category”
   2. “Excellent criteria that includes a sufficient number of examples to make this criteria far more accessible.”
   3. “some symptoms hold more weight and should be judged in the clinical context”
   4. “Will need to have movement disorders be a fixed criteria, our definitive cases have this”
   5. “Catatonia should be its own unique category”
   6. “Movement disorders should also be video taped to compare later on”
3. Category 3:
   1. “Have you had any cases of patients with EEG abnormalities alone without CSF findings and thought to be DSRD? what are rates of incidental EEG findings in Down syndrome? Is there baseline slowing even without regression? Is the epilepsy in patients with DS thought to be due to inflammation? I wonder if the criteria for probable could be either CSF or MRI abnormalities. It's also interesting that inflammation could be contributing to infantile spasms so wonder if there is a role for inflammation in patients with DS and IS.”
   2. “Not sure if this is defining a heterogenous disorder by including these”
   3. “Not always positive in all probable diagnoses”
   4. “I don't think this is essential for a diagnosis; clinical features alone may be informative”
   5. “Note that many will not have prior or baseline EEG. Findings of generalized slowing are common. Not sure if these are seen in catatonia. Are we excluding catatonia as a cause of regression?”
   6. “We need more data before including these”
   7. “1 and 2 may shed light on some cases of regression but should not be considered criteria for the syndrome itself. #3 can be seen in many adults w DS, regardless of whether they present regression and the role of EEG in increasing likelihood ratio for the diagnosis of regression is far from clear”
   8. “I don’t think I can recommend this for inclusion until more data is obtained. Would consider in the next iteration”
4. Category 4:
   1. “Would strongly recommend psychiatry evaluation if prominent catatonia, anxiety, depression symptoms to help rule out primary psychiatric disease”
   2. “Also medication effects”
   3. “some conditions may be obviously not regression, but it may not be possible to know at one time point of assessment”
   4. “Catatonia should not be considered exclusionary.”
   5. “should consider age, too”
